# Isolation and Characterisation of the *Bundooravirus* Genus and Phylogenetic Investigation of the *Salasmaviridae* Bacteriophages

**DOI:** 10.3390/v13081557

**Published:** 2021-08-06

**Authors:** Cassandra R. Stanton, Daniel T. F. Rice, Michael Beer, Steven Batinovic, Steve Petrovski

**Affiliations:** 1 Department of Physiology, Anatomy & Microbiology, La Trobe University, Melbourne, VIC 3086, Australia; c.stanton@latrobe.edu.au (C.R.S.); dtrice1@gmail.com (D.T.F.R.); s.batinovic@latrobe.edu.au (S.B.); 2 Department of Defence Science and Technology, Port Melbourne, VIC 3207, Australia; michael.beer@dst.defence.gov.au

**Keywords:** *Bacillus*, bacteriophage, phage, phi29-like, *Salasmaviridae*, phylogenetics

## Abstract

*Bacillus* is a highly diverse genus containing over 200 species that can be problematic in both industrial and medical settings. This is mainly attributed to *Bacillus* sp. being intrinsically resistant to an array of antimicrobial compounds, hence alternative treatment options are needed. In this study, two bacteriophages, PumA1 and PumA2 were isolated and characterized. Genome nucleotide analysis identified the two phages as novel at the DNA sequence level but contained proteins similar to phi29 and other related phages. Whole genome phylogenetic investigation of 34 phi29-like phages resulted in the formation of seven clusters that aligned with recent ICTV classifications. PumA1 and PumA2 share high genetic mosaicism and form a genus with another phage named WhyPhy, more recently isolated from the United States of America. The three phages within this cluster are the only candidates to infect *B. pumilus*. Sequence analysis of *B. pumilus* phage resistant mutants revealed that PumA1 and PumA2 require polymerized and peptidoglycan bound wall teichoic acid (WTA) for their infection. Bacteriophage classification is continuously evolving with the increasing phages’ sequences in public databases. Understanding phage evolution by utilizing a combination of phylogenetic approaches provides invaluable information as phages become legitimate alternatives in both human health and industrial processes.

## 1. Introduction

Organisms that belong to the *Bacillus* genus are Gram-positive, aerobic, endospore forming rods [1]. They are diverse and important environmental microbes; however, members of this genus have been implicated with human disease [2,3]. These organisms most commonly include *Bacillus cereus* and *Bacillus anthracis*, which are capable of causing severe foodborne illnesses and anthrax, respectively [4]. *Bacillus pumilus* has also been known to intermittently cause food borne illness and contamination in assumed sterile areas [5]. This is largely due to the ability of *B. pumilus* endospores to survive extreme environments, including hydrogen peroxide treatment, which is a common method of equipment sterilization [6]. *B. pumilus* genomes also contain an arsenal of genes able to survive oxidative stress and antimicrobial compounds [7]. This alarming robustness, along with the looming threat of antibiotic resistance, suggests that alternative treatments to chemical sterilization and antibiotics are required.

Bacteriophages (or phages) are viruses that propagate by infecting and lysing bacterial cells. Phages are predicted to be the most diverse and abundant biological entities on the planet [8,9]. Due to their ability to alter bacterial genomes through horizontal gene transfer and impact the population dynamics within microbial communities, they play a major role in microbial ecology and evolution [10,11]. Phages have also gained great interest as potential therapeutic agents to be used as an alternative to antibiotics [12,13]. With the emergence of next generation sequencing, an abundance of phage sequences are continuously deposited into public databases, providing comprehensive information on phage genetic diversity and taxonomy [14,15,16,17]. The abundance of sequence data has led to the creation of the Mycobacteriophage databases that has subsequently become the Actinobacteriophage database (phagesDB.org) [18]. This website has expanded to also include a *Bacillus* phage database with over 1400 *Bacillus* phage sequences uploaded. This useful resource has provided us with information on the genetic diversity and evolution of *Bacillus* phages. It is becoming increasingly important that the more *Bacillus* phage genome sequences we have, the better we understand their evolution and interactions with the environment and their hosts [19]. 

Grose et al. [20] conducted a thorough analysis of 93 *Bacillus* phage sequences and generated clusters based on genomic similarities. The groupings were based on that described previously for the *Mycobacterium* phages [21] and the phi29-like phages [22]. Cluster B was noted for phages relating to phi29, now classified in the *Salasvirus* genus. At the time, a small subset of phage sequences was available and was classified into three sub-clusters, including phi29 and PZA (grouped in sub-cluster B1), B103 and Nf (sub-cluster B2), and GA-1 (sub-cluster B3) [20]. In 2018, Schilling, Hoppert, and Hertel [9] discussed 21 phi29-like phage sequences available in GenBank, providing preliminary insights into this group’s genetic relatedness [9]. However, due to advances in viral classification techniques and the addition of other phi29-like phages in Genbank, this work is outdated. Recently, Li, et al. [23] isolated one of the newest members of this group, DLc1, and concluded that the now present 30 phi29-like phages should be included within the *Salasvirus* genus. However, this study failed to use multiple reticulate phylogeny methods for this classification and relied only on basic genome comparison methods. As of earlier this year, the International Committee on Virus Taxonomy (ICTV) reviewed the phi29-like phages and classified them in the new *Salasmaviridae* family [24]. However, within this report there were no detailed conclusions about these new taxonomic rankings, therefore, validation of this work is needed. 

In this study we isolated two novel *B. pumilus* phages, PumA1 and PumA2, from Australian soil samples. These phages were characterized based on morphology, host range, and genome sequence. Annotation and sequence analysis revealed that both phages are novel at the DNA sequence but share conserved protein families, genome structure, and phenotypic characteristics similar to phi29 and other *Salasmaviridae* phages. This study also provides an in-depth genomic analysis of the newly classified *Salasmaviridae* phages and insights into their evolution and diversity.

## 2. Materials and Methods

### 2.1. Bacterial Strains and Media

In this study, *Bacillus pumilus* LTU1 strain was used, isolated by Dr Darryl Reanney, from a soil sample collected from Victoria, Australia. Bacterial cultures were grown on LB (1% tryptone (Oxoid, Adelaide, Australia), 0.5% yeast extract (Oxoid), and 1% sodium chloride (Sigma)) broth or agar (LB plus 1.4% agar (Oxoid)) at 28 °C. All chemicals were obtained from Sigma, Sydney, Australia) unless otherwise stated.

### 2.2. Isolation and Purification of Phages and DNA Extraction

Bacteriophages were isolated from soil samples taken from multiple points within 30 km of Darwin, Australia. The soil samples (1 g) were suspended in 2 mL of sterile water. The mixture was vortexed for 60 s and then centrifuged for 5 min before being filtered through a cellulose acetate membrane filter (0.2-µm pore size) to remove cells and other solid matter. Following incubations, the remaining bacterial cells were removed by centrifugation and filtration through a 0.2-µm cellulose acetate membrane filter. Lawn plates of *B. pumilus* were prepared and 20 µL aliquots of enriched supernatants were applied onto the lawn plates and allowed to dry. Plates were incubated overnight and visually inspected for the presence of the plaques the following day. Single plaques were observed and isolated from two different soil samples in the Northern Territory in Australia. Plaques were purified through six rounds of dilution and re-isolation to ensure their purity.

### 2.3. Phage DNA Isolation, Genome Sequencing, and Annotation

Purified phage particles were polyethylene glycol (PEG) precipitated followed by a proteinase K treatment to extract DNA as described previously [25]. Isolated phage DNA (100 ng) were prepared using the NEBNext^®^ Ultra™ II DNA Library Prep Kit (NEB) (Australia) followed by whole genome sequencing on an Illumina MiSeq v3 600-cycle kit with 300 bp paired-end reads. Raw data were filtered using Trim Galore v0.6.4 with the default settings (Q scores of ≥20, with automatic adapter detection) [26]. Phage genomes were assembled with SPAdes v3.12.0 with default settings. The genome termini were corrected upon manual inspection of raw sequencing reads using CLC Genomics Workbench v9.5.4 (Qiagen, Melbourne, Victoria, Australia).

Putative open reading frames (ORFs) were predicted using Glimmer v3 and manually confirmed [27]. Sequence similarity searches were conducted using the predicted amino acid sequences against the GenBank database and the BLASTP algorithm was used with an E-value significance cut off of 10^−4^ [28,29]. Conserved domains and motifs were identified using the conserved domain database (CDD) (http://www.ncbi.nlm.nih.gov/Structure/cdd/cdd.shtml) (accessed on 7 March 2019) and the Pfam database (http://pfam.sanger.ac.uk) (accessed on 7 March 2019) [30]. The presence of genes encoding tRNAs was screened for using ARAGORN (http://130.235.244.92/ARAGORN/) (accessed on 7 March 2019) [31]. 

### 2.4. Electron Microscopy

Copper grids (ProSciTech, Townsville, Queensland, Australia) coated with carbon and formvar were subjected to a glow discharge treatment for 60 s. A total of 20 µL of high titer (>10^9^ pfu/mL) phage filtrates were placed onto the grids, incubated for 10 min, and followed by removal of excess residue with filter paper. Grids were washed twice with 5 µL MilliQ, each wash being absorbed with filter paper. The grids were then negatively stained with 3 µL of 2% (*w*/*v*) uranyl acetate, followed by immediate removal with filter paper and one final MilliQ wash as outlined previously. The grids were then left to dry in a laminar flow for 30 min before imaging. The grids were examined under a JEOL JEM02010HC electron microscope.

### 2.5. Nucleotide Sequence

The nucleotide sequences for phages vB_BpuP_PumA1 and vB_BpuP_PumA2 have been deposited in GenBank under the accession numbers MN524844 and MN524845, respectively. 

### 2.6. Identification of Phage Resistant B. pumilus Mutants 

Lawn plates of *B. pumilus* and either PumA1 or PumA2 flooded in high titer were grown for 24 h. Colonies that emerged in the clearings were picked, streaked out for a total of three times, and re-spotted with phage to test that their resistance was stable. DNA extractions of the wild type *B. pumilus* and the strains showing phage resistance were prepared using the Wizard Genomic DNA Purification Kit (Promega, Sydney, Australia) as per manufacturer’s instructions. The DNA samples were then prepared for next generation sequencing as outlined above. For the wild type *B. pumilus*, the raw sequencing data was trimmed using Trim Galore v0.6.4 and the genome assembled using Unicycler v0.4.8. For SNP analysis, Snippy (Galaxy V.4.5.0) was used. The assembled wild type *B. pumilus* was the reference genome and each mutant strain was compared for differences. 

### 2.7. Whole Genome Analysis and Clustering

The NCBI and *Bacillus* Phage (phagesdb.org) Databases were examined for related phi29-like phages. Thirty-four complete phage sequences were found on either database and used for this study (Table 1). A dot plot using the Genome Pair Rapid Dotter program (GEPARD) was chosen as a preliminary guide for clustering [32]. The genome sequences were collated into one FASTA file in the order of nucleotide identity found through BLASTN results. Clusters were validated using VIRIDIC v1.0 software [33].

Whole genome alignments of all phages and their clusters were undertaken using BLASTn comparisons and visualized with Easyfig [34]. For gene content analysis, a Clustal Omega alignment was performed and visualized in Splitstree4 [35,36]. Using the Neighbor-Joining method, a consensus network tree was developed. To create the reticulate gene sharing network, vConTACT v2.0 0.9.17 [37], a gene mapping program, was used. Gene2Genome was first used to assign each protein coding sequence of each phage and map it to its contig/genome ID. This output file was then combined with the collated FASTA file previously used and ran in vConTACT v.2.0. The network output file of vConTACT 2v.2.0 was then visualized in Cytoscape v3.8.1 [38]. Finally, a core protein phylogeny tree was created using both the DNA polymerase and DNA encapsidation ATPase in VICTOR [39]. All pairwise comparisons of the nucleotide sequences were conducted using the Genome-BLAST Distance Phylogeny (GBDP) method and branch support was inferred from 100 pseudo-bootstrap replicates each. The tree was then visualized using iTOL v6.2 [40]. The tree was rooted at midpoint and meta-data overlayed.

## 3. Results 

### 3.1. Isolation of Bacteriophages and Their Morphological Features 

**Figure 1:**
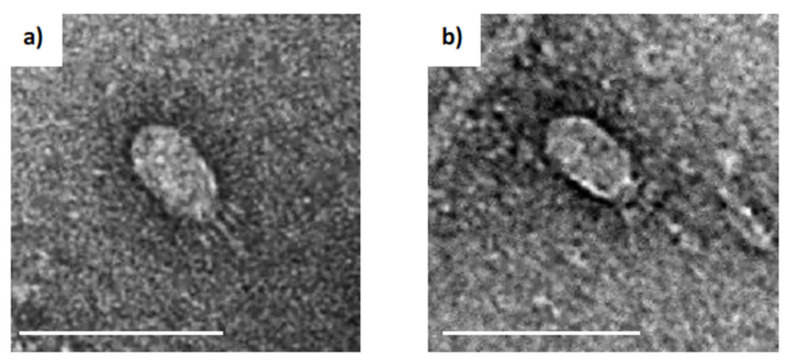
PumA1 and PumA2 morphologies. Transmission electron micrograph of (**a**) PumA1 and (**b**) PumA2. Scale bar = 100 nm.

After screening multiple soil samples collected in Darwin (Northern Territory, Australia), two samples from different locations produced plaques on lawn plates of *B. pumilus* (LTU1). They were isolated, purified, and named vB_BpuP_PumA1 (PumA1) and vB_BpuP_PumA2 (PumA2). For host range analysis, the phages were tested against other *Bacillus* species in our culture collection including *B. anthracis*, *B. subtilis, B. mycoides*, and *B. cereus*. Both phages exclusively lysed *B. pumilus,* suggesting a narrow host range (regarding available strains). Transmission electron microscopy imaging of negatively stained phages demonstrated that PumA1 and PumA2 displayed short tails and small, elongated capsids of 52 ± 5 nm (length) × 29 ± 6 nm (width) (Figure 1). 

### 3.2. Sequencing and Genomic Features of PumA1 and PumA2

**Figure 2:**
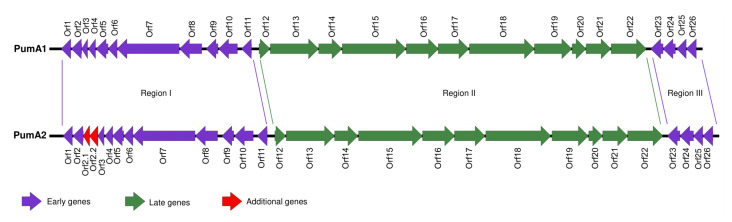
Genome maps of PumA1 and PumA2. Colored arrows represent open reading frames (ORFs). Purple arrows show the early region genes (regions I and II), and the green arrows depict the late region genes (region II). Key genes are named and full descriptions of the ORF functions are in Table S1.

PumA1 and PumA2 were sequenced using Illumina sequencing technology. Genome assembly revealed both phages had linear genomes of 18,466 bp and 18,932 bp, respectively. Annotation of the PumA1 and PumA2 genomes revealed that they contained 26 and 28 putative open reading frames, respectively, and no tRNA genes (Figure 2). The genomes of both PumA1 and PumA2 share 82% similarity over 98% of the genomes. When compared to DNA sequences in the GenBank database, both phages are unique, with only 4% to 20% of the genomes sharing between 65% and 73% sequence identities with other phi29-like phage genomes. The phage genomes were flanked by 11 bp inverted repeat sequences (5′-AATGTAAAGGT-3′) consistent with phi29-like phages that all contain variations of inverted repeats [53]. The predicted amino acid sequence of each open reading frame was used in a BLASTp analysis to determine the closest homologue. Predicted functions can be assigned to sixteen gene products (Table S1). The genes were numbered consecutively with the exceptions of *orf2.1* and *orf2.2*, which are present in PumA2 but not in PumA1. The genome organization and structure are similar to that of phage phi29 and its relatives, and the gene products share conserved similarities at the amino acid level [54].

The genomes of both PumA1 and PumA2 can be separated into three different modules based on the direction the genes are transcribed (Regions I, II, and III) (Figure 2). Regions I and III (also known as the early genes) contain genes that are transcribed in the same direction and are located at the 5′ and 3′ ends of the genomes. Region II (also referred to as the late genes) is located in the center of the genome and is transcribed in the opposite direction to the other genes [9]. 

Region I of the genomes encompasses eleven genes for PumA1 and thirteen genes for PumA2. The proteins encoded by the open reading frames *orf1-orf6* in both phage genomes have no known predicted function and are noted as hypothetical proteins. However, *orf7* to *orf11* are described and associated with the phi29-like phages [9,44,45]. The genes encoding the DNA polymerase (*orf7*) are highly conserved between the phi29-like phages, and when compared using BLASTn, most of this gene (83% coverage) is 65% homologous to the DNA polymerase of phage phi29. The terminal binding protein in PumA1 and PumA2 is encoded by *orf8*, adjacent to the DNA polymerase [55]. The remaining genes within the first region encode a DNA transcriptional activator for the late genes (*orf9*), containing a characteristic conserved motif pfam05464 similar to that observed in phi29 [56,57], followed by a gene encoding single stranded binding protein (*orf10*) and double stranded binding protein (*orf11*). Region III contains four genes (*orf23-orf26*) transcribed in the same direction as the genes in region I. Three of the genes have unknown functions, and *orf24* encodes a DNA replication organizer with a pfam06720 motif. 

Region II contains genes *orf12-orf22* that encode structural and morphogenesis genes. The head morphogenesis protein, *orf12*, contains a pfam11418 motif, similar to phi29 scaffolding protein [58]. This is followed by *orf13*, a putative major head protein containing a bacterial Ig-like domain (pfam02368) and *orf14*, a head fiber protein and motif (pfam11133). The major tail protein (*orf15*) contains a pfam16838 motif, conserved across groups of podoviruses [50,59]. The proteins that connect the phage head and tail are encoded by *orf16* containing a pfam05352 [60], *orf17* encoding a lower collar protein with the PHA00148 motif [61,62], and *orf18* encoding a minor structural protein that is suspected to form the pre-neck appendage protein. *Orf18*’s closest homologues are found in other phi29-like phages WhyPhy and SRT01hs and a *Staphylococcus* phage ST134 with a conserved motif TIGR04523. This is followed by another morphogenesis protein, *orf19*, with the characteristic motif pfam01551. A putative holin is encoded by *orf20* with the pfam05105 motif conserved in bacteriophage holin proteins. *Orf21* encodes an endolysin with two motifs, pfam01520 and pfam01476 [63]. The last gene in the module *orf22* encodes a protein that is predicted to encode a podovirus DNA encapsidation protein with the characteristic motif pfam05894 [64]. 

### 3.3. Whole Genome Comparison and Clustering of phi29-Like Phages 

Since the genome organization and protein homologies of PumA1 and PumA2 were reminiscent of phi29, we next investigated the evolutionary relationship shared between PumA1, PumA2, and other phi29-like phages. The GenBank and *Bacillus* phage databases were searched for complete phage genomes that shared genomic similarities to phi29. This led to the identification of 34 phages, including the two isolated in this study (Table 1). Hatfull, et al. [65] previously described a classification system using a four-method approach to cluster 60 mycobacteriophage genomes. We employed their approach as a guide and included whole genome comparisons, network and clustering analysis, and candidate gene phylogenetics to organize the phi29-like phages into respective clusters (Figure S1). The clusters were expanded from the ones previously described by Grose, Jensen, Burnett, and Breakwell [20] and employ the same naming style of B and subclusters numbered (e.g., B1). The number of clusters were expanded from three [20] to seven and three singleton phages. New members were added to existing clusters and align with the ICTV classifications. While no new clusters or potential genera were formed from this analysis, this study provides a justification for the ICTV taxonomic rankings and framework for future clustering and classification of phages. 

### 3.4. Dot Plot Analysis and Genomic Identities

**Figure 3:**
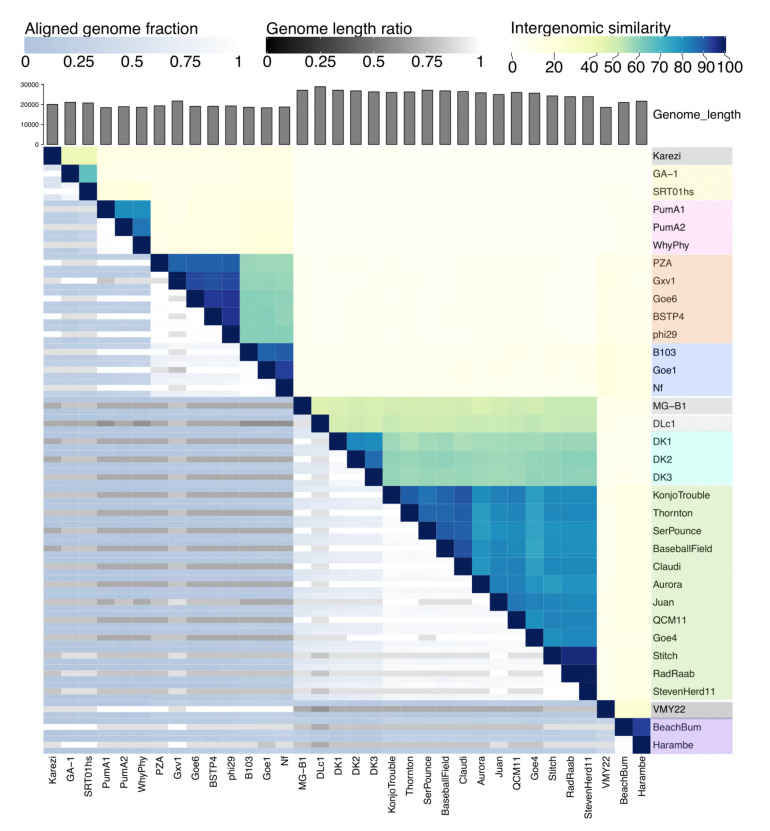
VIRIDIC heatmap of the 34 phi29-like phages. Intergenomic similarities of the phages are shown on the right side with the colored scale where more defined clusters are seen compared to the dot plot. The similarities scores are found in Table S2. The aligned genome fraction and genome length ratio values are shown on the left side with their corresponding scales. Overlayed is the color coding of the seven major clusters, where grey indicates singleton phages.

Dot plot analysis of the 34 genomes revealed six clusters and two phages not pertaining to a cluster, referred to as singletons (Figure S2). To further define the clusters and provide a numerical value to their similarities, we used the Virus Intergenomic Distance Calculator (VIRIDIC) to calculate intergenomic similarities between each phage. VIRIDIC combines several similarity algorithms with genome alignment and length ratios to capture overall relatedness of prokaryotic viruses [33]. In correlation with the dot plot analysis, the heatmap presents the 34 phages in the same clustering pattern (Figure 3). Each cluster shares similarities of between 65.65–99.87% (Table S2). The clusters are now referred to as from B1 to B7, expanding from previous clusters [20].

### 3.5. Genome Map Alignments

**Figure 4:**
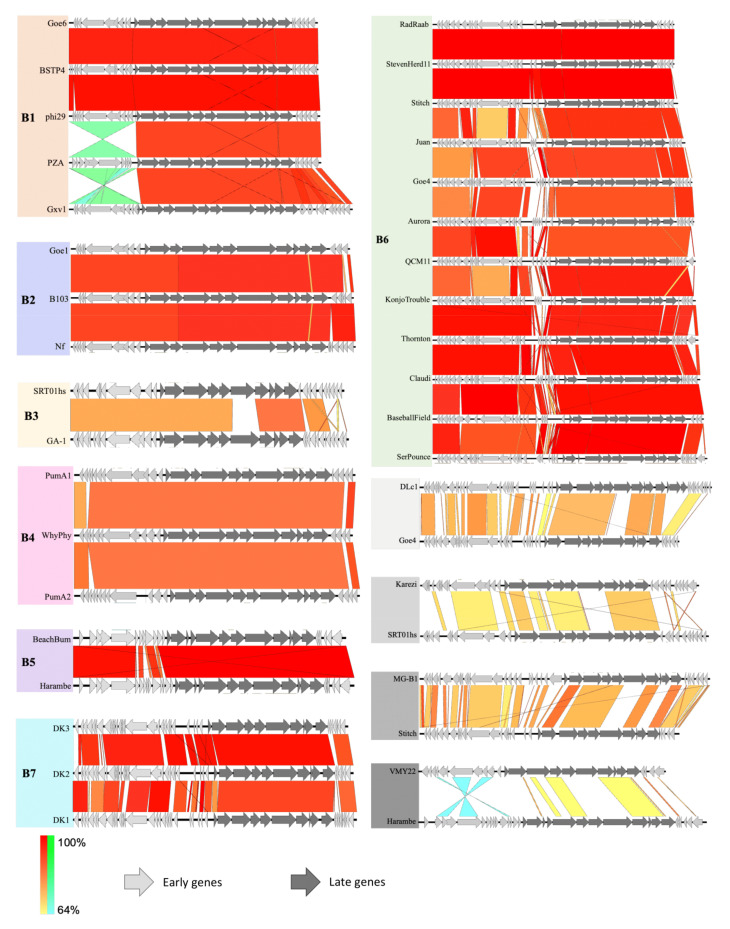
Genome map alignments of each cluster and singletons (shaded grey) with their closest relating phage. Color bar ranges from red to yellow, showing identities, and the blue to green shows homology in any inverted sections.

Each cluster was aligned to show the dissimilarities between genomes (Figure 4). PumA1 and PumA2 contain an extra 796 bp and 1240 bp respectively compared to the ancestral phage phi29 in the 5′ early gene region. These hyperplastic regions are common throughout the phi29-like phages, where large insertions of up to 8256 bp are seen in the largest phi29-like phage, DLc1. Each cluster also shares the same pattern of insertions between them. The additional genes have no known functions but are presumed to be involved in the infection or replication processes since they are located within that region. Interestingly, the phages with genome sizes over 20 kb no longer contain the head fiber gene, a characteristic feature of phi29. The singletons show the least similarity to phi29 and other phages in this group, with small regions of similarity to their closest related phage. 

### 3.6. Gene Sharing Networks

**Figure 5:**
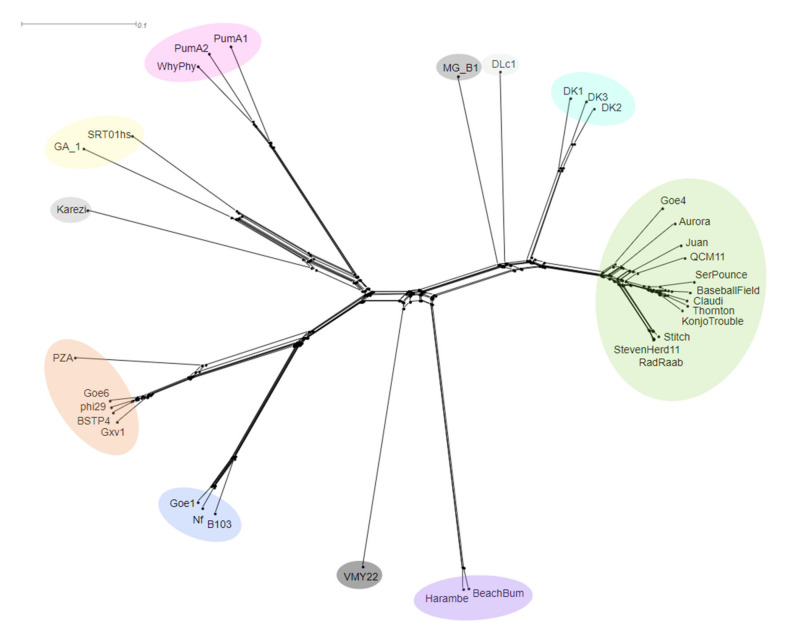
Neighbor-Joining network of the phi29-like phages. Clustal Omega alignments of the whole genomes were calculated and visualized in Splitstree. Clusters are displayed by corresponding colored overlays.

Reticulate networks have been recently shown to provide an accurate representation of phage relatedness versus traditional rooted phylogenetics, since phages undergo many recombination and horizontal gene transfer events [66]. To test if these methods aligned with the comparison methods previously mentioned, two reticulate methods were used. Firstly, an unrooted phylogenetic network was created in Splitstree4 using whole genome CLUSTAL Omega alignment (Figure 5). This network showed a consistent pattern of clustering in agreement with the other techniques. 

**Figure 6:**
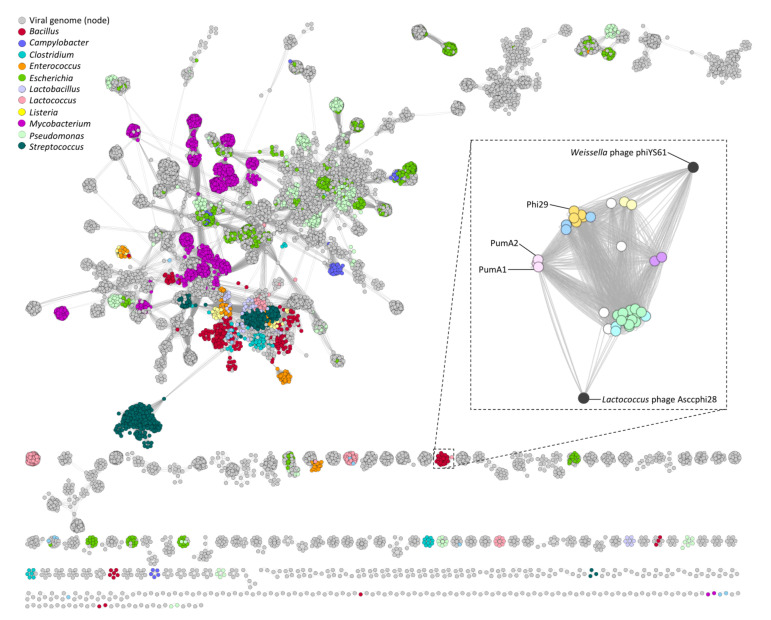
vConTACT2 reticulate genome network of phages in the Genbank and ICTV databases. A “node” or singular phage genome is connected by an “edge” or line which is calculated by how many proteins they share and is scored by significance. The *Salasmaviridae*/phi29-like phages cluster was extracted in the dotted box. The network is displayed using an edge-weight spring embedded layout that repels phages based on their lack of similarity in protein cluster scores.

vConTACT v.2.0 was then used in conjunction with the Splitstree network. vConTACT v.2.0 is a newly developed software for virus classification that extracts, aligns, and clusters all predicted input proteins [37]. The protein clusters are then used to calculate viral clusters (VC) by am “edge” weight or statistical confidence due to the amount of protein clusters that each phage shares. This is compared to a global network of phages in the GenBank database. Figure 6 depicts the global network produced from vConTACT v.2.0 with various phages color coded by their host genera. The phi29-like phages are not connected to the main network, showing little gene-sharing outside of this group of phages. When the phi29-like network is expanded, there is a clear differentiation of each phage cluster, denoted by the individual cluster colors. While these phages do not connect to the main network, they share proteins with two other phages that are not infective for *Bacillus*, *Lactococcus* phage Asccphi28 [67] and *Weisella* phage phiYS61 [68], which are genotypically similar to the phi29-like phages. This form of reticulate phylogenetics helped corroborate the established clusters previously outlined and is an accurate tool for investigating phage gene-sharing and evolution. 

### 3.7. Candidate Gene Analysis 

**Figure 7:**
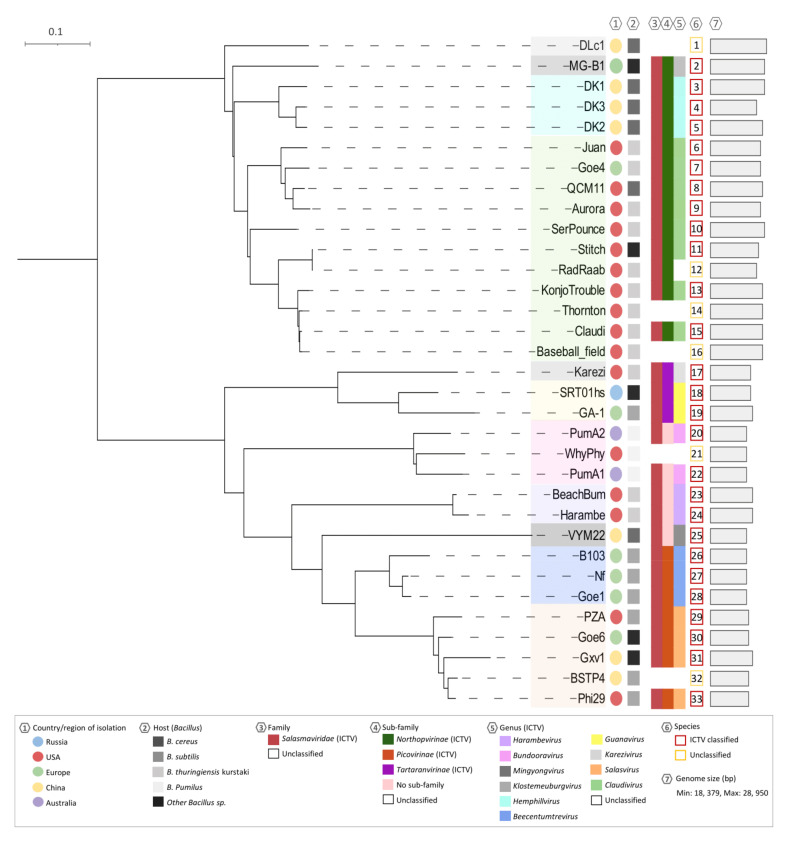
Phylogenetic tree using alignments of both DNA polymerase and encapsidation ATPase as candidate genes. The tree is rooted at midpoint and branches are supported by 100 bootstrap replicates. The colored shadings over each phage name indicate the clustering based off the other genomic comparisons; other metadata is defined in the legend. This clustering and color coding also correlate to the ICTV genus classifications. StevenHerd11 was eliminated from this tree due to its almost identical similarity to RadRaab.

Finally, a phylogenetic tree was created combining two conserved and integral genes, DNA polymerase and DNA encapsidation ATPase (Figure 7). The tree correlates with the whole genome comparison approaches as the phages are seen to group with their clusters. The tree splits into two distinctive branches with singletons DLc1 and MG-B1 and clusters B6 and B7 diverging from the rest of the clusters. This correlates with the genome sizes of these phages, as they are the largest genomes of the phi29-like group. The branch distances are also short within the clusters, particularly B6 members, signifying small base pair substitutions. The other factors that are seen to contribute to the phages’ evolution are host species and country or region they were isolated. These clusters also agree with the ICTV taxonomic rankings as outlined by metadata on the tree.

### 3.8. PumA1 and PumA2 Host Receptor Site 

PumA1 and PumA2 displayed a narrow host range, only able to infect the *B. pumilus* strain in our collection. This appears to be a characteristic of the B4 cluster or *Bundooravirus* genus. It has been shown that phi29 requires polymerized teichoic acid for its attachment to *B. subtilis* [69]. Given the observed host range differences between phi29 and the two phages isolated in this study, we pursued an investigation into the host receptor of PumA1 and PumA2. *B. pumilus* colonies that developed stable resistance to PumA1 and PumA2 were isolated, and whole genome sequenced to determine which genomic modifications were causing resistance to phages. Mutations were found in either the *tagF* or *tagT* genes, which are a part of the teichoic acid synthesis operon [70,71,72]. Four of the seven variant strains isolated had modifications to the *tagF* gene, and the other three had mutations in the *tagT* gene (Table S3). The majority of the mutants (A2M1, A2M11, A1M3, A2M14, and A1M5) contained frameshift mutations resulting in early termination of protein translation. Most of these frameshifts occurred near the N-terminus of the respective proteins likely resulting in non-functional TagT or TagF proteins. The remaining mutant had a single amino acid substitution in TagF (G688S). It is unclear how this mutation affects protein structure and function.

## 4. Discussion

Advances in whole genome sequencing techniques and the rise of antibiotic resistance has resulted in an abundance of publicly available sequencing data and reinvigoration of phage-based studies. In this study, we isolated two novel, narrow host range *Bacillus pumilus* phages, PumA1 and PumA2. Both phages were unable to form plaques on *B. pumilus* strains that had mutations in the *tagT* or *tagF* genes. Wall teichoic acid (WTA) is a major component of Gram-positive cell walls, with the *tag* operons encoding the necessary machine for its synthesis [70,71]. TagF is a poly(glycerol phosphate) polymerase that plays a key role in the formation of the glycerol phosphate chains in WTA. Single nucleotide polymorphisms have been shown to significantly alter the function of TagF, resulting in decreased polymerase activity [71]. Whereas *tagT*, which encodes a LytR-CspA-Psr (LCP) family protein, is implicated in the final stage of WTA synthesis, in which it catalyzes the attachment between the teichoic acid polymers and peptidoglycan. In *B. subtilis* mutants in which the *tagTUV* operon was knocked out, cells appear more rounded and lack the typical rod shape and teichoic acid present in growth cultures but are detached from peptidoglycan [70]. Given that both phages studied here and phi29 appear to share the same surface receptor, we hypothesize that the *Bundooravirus* phages have differentiated tail structures to specifically detect *B. pumilus* teichoic acid. 

The *Salasmaviridae* (or phi29-like) group contain the smallest known genome sizes of phages that infect *Bacillus* sp. The highly conserved phages of this family are distributed throughout several continents, with PumA1 and PumA2 currently the only phages isolated from Australia. The multi-approach clustering indicated a correlation of *Salasmaviridae* phages by host range and genome size. This was aptly demonstrated with the *Bundooravirus* phages isolated in this study, which appeared to have a narrow host range against *B. pumilus*. We also noticed a correlation of cluster formation with the geographical location of phage isolation. This pattern is likely driven by the host bacterium’s biogeography and population; this may become clearer as more *Salasmaviridae* phages are isolated and sequenced [72,73].

Since the *Salasmaviridae* phages follow a strict lytic lifecycle with no evidence of lysogenic activity, there may be low gene-content flux and recombination event rates between themselves, their hosts, and other phages [74]. However, environmental and host pressures can naturally result in genomic mutations, and as a phage gains new adaptations such as expanded host range, this can result in enough genetic variance to be included as a new species and genus [19,66,75]. While the phage genomes remain well conserved in their arrangement and modules of essential genes, there appears to be three main regions where insertions are present, all in the “early” replication gene modules. These additional genes correlate to increased genome size and how the phages cluster, reminiscent of hyperplastic regions seen in other groups of phages [76]. While most of these early genes have unknown functions, it is hypothesized that early proteins are associated with phage-host interactions. This includes genes that encode for protection from host degradation and restriction, anti-CRISPR, and inhibition of host transcription [77]. Though they are not essential for phage function, and can be lost and gained readily, they have the potential to be advantageous for adaption to their host [78]. Mutations leading to increased genome and capsid sizes tend to be more favorable and have been conserved throughout the *Northopvirinae* phages [79]. In contrast, the structural or “late” gene modules remain almost identical in arrangement and constitution throughout the *Salasmaviridae* phages. One notable difference seen in the “late genes” region is that any phage with a genome larger than 20 kb had lost the “head fiber” gene. The head fibers are involved in sensing and interact with the bacteria cell wall but are not essential for phage viability [80,81]. Phages that have acquired new genes are seen to have an enlarged capsid and altered capsid architecture in response [79]. It is hypothesized that since the fibers have no essential functions and the addition of newer genes has either forced or allowed these phages to expand their capsids, the head fibers are no longer able to attach to this new capsid structure. Nevertheless, this needs to be investigated further. 

## 5. Conclusions

PumA1 and PumA2 represent a novel genus in the newly formed *Salasmaviridae* family, *Bundooravirus*. Multiple clustering approaches, including reticulate networks, resulted in the clustering of all current phi29-like phages, including those recently classified by the ICTV. This clustering agrees with taxonomic rankings and allows for the addition of several phages into this family. Thornton and Baseball_field should be included in the *Claudivirus* genus, BSTP4 should be classified in the *Salasvirus* genus, and DLc1 should be classified into the *Northopvirinae* sub-family but in its own genus. WhyPhy should be classified into the *Bundooravirus* genus with PumA1 and PumA2. Current data suggests the *Bundooravirus* phages are unique in their host range and require *B. pumilus* specific teichoic acid residues for infection. The *Salasmaviridae* (or phi29-like) phages are globally distributed but remain well conserved in genome organization and protein domains. However, there are three patterns of clustering that contribute to their evolution and classification, including geographical location, host range, and genome size. This study demonstrated that a combination of whole genome comparisons and rooted and reticulate phylogenetic models can be used to our advantage to order and classify phages. Understanding phage evolution and their relationships with other phages and the environment will provide us with invaluable information into phage phylogenetics as their usage in medical and industrial processes continues.

## Figures and Tables

**Table 1:** Current *Salasmaviridae* (phi29-like) phages that have been sequenced and submitted to the Genbank and *Bacillus* phage database as of February 2021.

Bacteriophage	Country Isolated	Accession Number	Year Isolated	Genome Size (kb)	Reference	Host
**PumA1**	Australia	MN524844	2017	18,446	This study	*B. pumilus*
**PumA2**	Australia	MN524845	2017	18,932	This study	*B. pumilus*
**MG-B1**	Austria	KC685370	2011	27,190	[41]	*B. weihenstephanensis*
**VMY22**	China	KT780304	2015	18,609	[42]	*B. cereus*
**Gxv1**	China	MT459794	2020	21,781	[43]	*Bacillus* sp.
**DK2**	China	MK284527	2018	23,357	[44]	*B. cereus*
**DK3**	China	MK284528	2018	26,865	[44]	*B. cereus*
**DK1**	China	MK284526	2018	27,180	[44]	*B. cereus*
**DLc1**	China	MW012634	2020	28,950	[23]	*B. cereus*
**Goe1**	Germany	KU831549	2014	18,379	[45]	*B. subtilis*
**Goe6**	Germany	MF407276	2017	19,105	Unpublished	*B. velezensis*
**Goe4**	Germany	MH817022	2018	25,722	[9]	*B. thuringiensis* kurstaki
**B103**	Prague	X99260	1981	18,630	[46]	*B. subtilis*
**SRT01hs**	Russia	MN857617	2020	20,784	Unpublished	*B. altitudinis*
**GA-1**	Scotland	X96987	1965	21,129	[47]	*B. subtilis*
**BSTP4**	South Korea	MW354668	2020	19,145	Unpublished	*B. subtilis*
**Nf**	Spain	EU622808	2008	18,753	[48]	*B. subtilis*
**PZA**	USA	PZACG	1976	19,366	[49]	*B. subtilis*
**Phi29**	USA	EU771092	1965	19,828	[50]	*B. subtilis*
**Karezi**	USA	MN082625	2013	20,083	Unpublished	*B. thuringiensis* kurstaki
**BeachBum**	USA	KY921761	2016	21,054	[51]	*B. thuringiensis* kutstaki
**Harambe**	USA	KY821088	2016	21,684	[51]	*B. thuringiensis* kutstaki
**RadRaab**	USA	MF156580	2016	23,946	Unpublished	*B. thuringiensis* kurstaki
**StevenHerd11**	USA	MK084630	2017	23,953	Unpublished	*B. thuringiensis* kurstaki
**Stitch**	USA	KX349901	2012	24,320	Unpublished	*Bacillus* sp.
**Juan**	USA	MF156577	2016	25,032	Unpublished	*B. thuringiensis* kurstaki
**Aurora**	USA	KX349899	2010	25,908	Unpublished	*B. thuringiensis* kurstaki
**QCM11**	USA	KX961631	2016	26,054	Unpublished	*B. cereus* group
**KonjoTrouble**	USA	MF156578	2016	26,061	Unpublished	*B. thuringiensis* kurstaki
**Claudi**	USA	KX349900	2014	26,504	Unpublished	*B. thuringiensis* kurstaki
**SerPounce**	USA	KY947509	2016	27,206	[51]	*B. thuringiensis* kurstaki
**WhyPhy**	USA	MW419775	2020	18,642	Unpublished	*B. pumilus*
**Thornton**	USA	MW348917	2017	26,319	Unpublished	*B. thuringiensis* kurstaki
**Baseball_field**	USA	MT777452	2015	26, 863	[52]	*B. thuringiensis* kurstaki

## Data Availability

The nucleotide sequences for phages vB_BpuP_PumA1 and vB_BpuP_PumA2 have been deposited in GenBank under the accession numbers MN524844 and MN524845, respectively.

## Supplementary Materials

The following are available online at https://www.mdpi.com/article/10.3390/v13081557/s1, Figure S1: Workflow for whole genome comparisons; Figure S2: Whole genome dot plot of the *Salasmaviridae*/phi29-like phages; Table S1: PumA1 and PumA2 genome annotations; Table S2: VIRDIC intergenomic similarities between clusters; Table S3: *B. pumilus* mutant strains resistant to PumA1 and PumA2 infection.
