# Supplementary material for: Isolation and Characterisation of the Bundooravirus Genus and Phylogenetic Investigation of the Salasmaviridae Bacteriophages
Source: Viruses. 2021 Aug 6;13(8):1557. doi: 10.3390/v13081557 (PMC8402886; doi:10.3390/v13081557)
Supplement: Supplementary file 1 [file viruses-13-01557-s001.zip › viruses-1297560-SI.pdf]

Table S1. PumA1 and PumA2 genome annotations

PumA1	Genome coordinates	Length (bp)	Direction	Product
**Orf1**	336 - 608	273	Reverse	Hypothetical protein
**Orf2**	621 - 917	297	Reverse	Hypothetical protein
**Orf3**	914 - 1084	171	Reverse	Hypothetical protein
**Orf4**	1101 - 1307	207	Reverse	Hypothetical protein
**Orf5**	1318 - 1659	342	Reverse	Hypothetical protein
**Orf6**	1656 - 1919	264	Reverse	Hypothetical protein
**Orf7**	1912 - 3672	1761	Reverse	DNA polymerase
**Orf8**	3678 - 4310	633	Reverse	Terminal protein
**Orf9**	4427 - 4768	342	Reverse	Transcriptional activator
**Orf10**	4785 - 5318	534	Reverse	ssDNA binding protein
**Orf11**	5426 - 5716	291	Reverse	Hypothetical protein
**Orf12**	5926 - 6240	315	Forward	Head morphogenesis protein
**Orf13**	6247 - 7614	1368	Forward	Major head protein
**Orf14**	7614 - 8276	663	Forward	Head fibre protein
**Orf15**	8280 - 10085	1806	Forward	Tail protein
**Orf16**	10087 - 11001	915	Forward	Upper collar protein
**Orf17**	10991 - 11860	870	Forward	Lower collar protein
**Orf18**	11873 - 13714	1842	Forward	Minor structural protein
**Orf19**	13711 - 14769	1059	Forward	Morphogenesis protein
**Orf20**	14776 - 15174	399	Forward	Holin
**Orf21**	15171 - 15878	708	Forward	Endolysin
**Orf22**	15878 - 16876	999	Forward	Encapsidation protein
**Orf23**	16996 - 17346	351	Reverse	Hypothetical protein
**Orf24**	17343 - 17696	354	Reverse	DNA replication organiser
**Orf25**	17732 - 18001	270	Reverse	Hypothetical protein
**Orf26**	18001 - 18288	288	Reverse	Hypothetical protein
**PumA2**	**Genome coordinates**	**Length (bp)**	**Direction**	**Product**
**Orf1**	384 - 653	270	Reverse	Hypothetical protein
**Orf2**	666 - 962	297	Reverse	Hypothetical protein
**Orf2.1**	959 - 1141	183	Reverse	Hypothetical protein

**Orf2.2**	1148 - 1375	228	Reverse	Hypothetical protein
**Orf3**	1372 - 1545	174	Reverse	Hypothetical protein
**Orf4**	1568 - 1792	225	Reverse	Hypothetical protein
**Orf5**	1779 - 2105	327	Reverse	Hypothetical protein
**Orf6**	2102 - 2365	264	Reverse	Hypothetical protein
**Orf7**	2358 - 4118	1761	Reverse	DNA polymerase
**Orf8**	4125 - 4757	633	Reverse	Terminal protein
**Orf9**	4874 - 5215	342	Reverse	Transcriptional activator
**Orf10**	5232 - 5765	534	Reverse	ssDNA binding protein
**Orf11**	5871 - 6161	291	Reverse	Hypothetical protein
**Orf12**	6373 - 6690	318	Forward	Head morphogenesis protein
**Orf13**	6697 - 8064	1368	Forward	Major head protein
**Orf14**	8064 - 8726	663	Forward	Head fibre protein
**Orf15**	8741 - 10546	1806	Forward	Tail protein
**Orf16**	10547 - 11461	915	Forward	Upper collar protein
**Orf17**	11451 - 12320	870	Forward	Lower collar protein
**Orf18**	12336 - 14180	1845	Forward	Minor structural protein
**Orf19**	14207 - 15238	1032	Forward	Morphogenesis protein
**Orf20**	15246 - 15644	399	Forward	Holin
**Orf21**	15641 - 16348	708	Forward	Endolysin
**Orf22**	16348 - 17346	999	Forward	Encapsidation protein
**Orf23**	17466 - 17819	354	Reverse	Hypothetical protein
**Orf24**	17816 - 18208	393	Reverse	DNA replication organiser
**Orf25**	18205 - 18468	264	Reverse	Hypothetical protein
**Orf26**	18468 - 18755	288	Reverse	Hypothetical protein

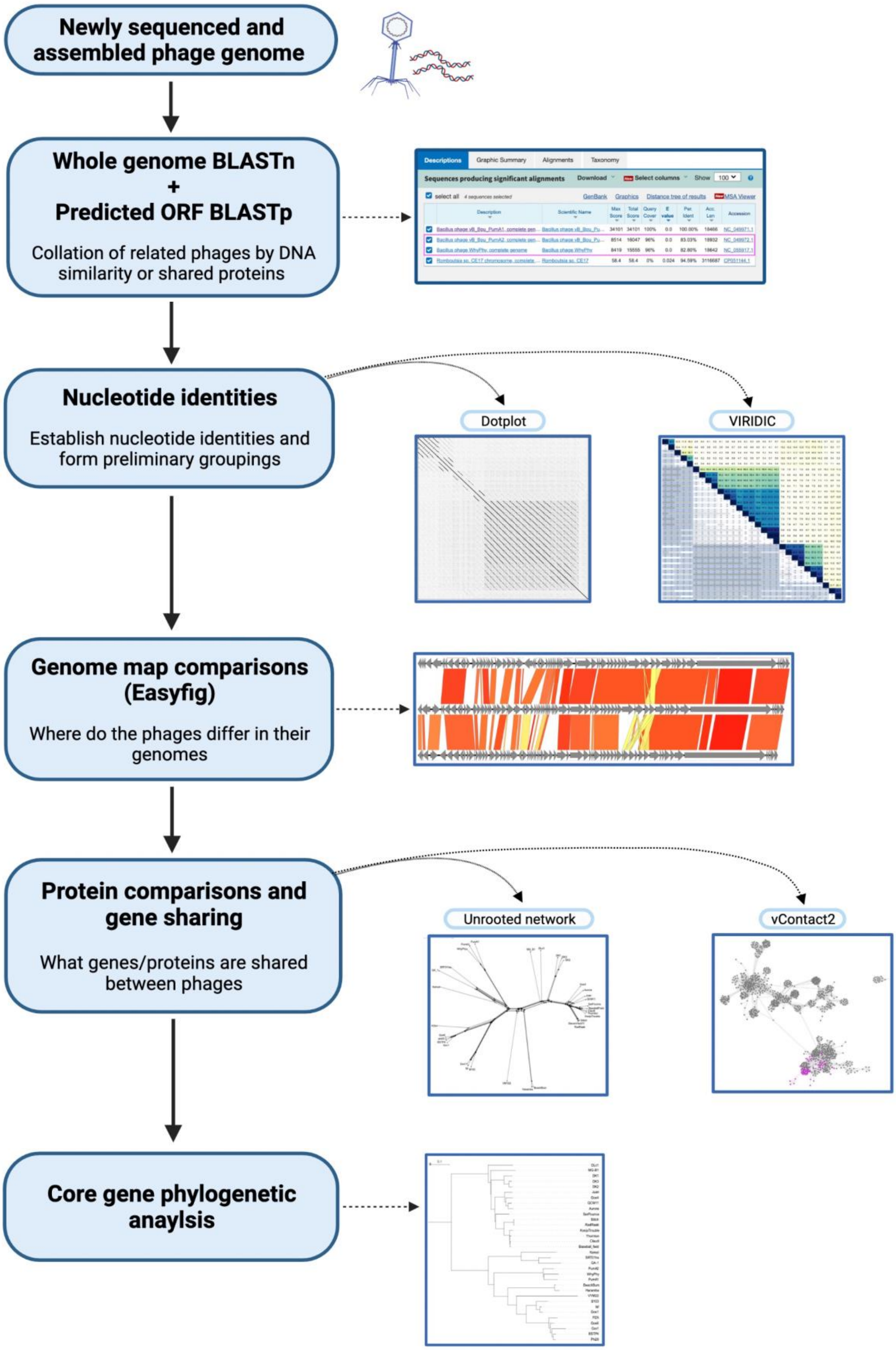

Figure S1. Workflow for the whole genome comparisons. These methods provide a robust and detailed examination of phage phylogenetics that takes into account their higher rates of gene sharing.

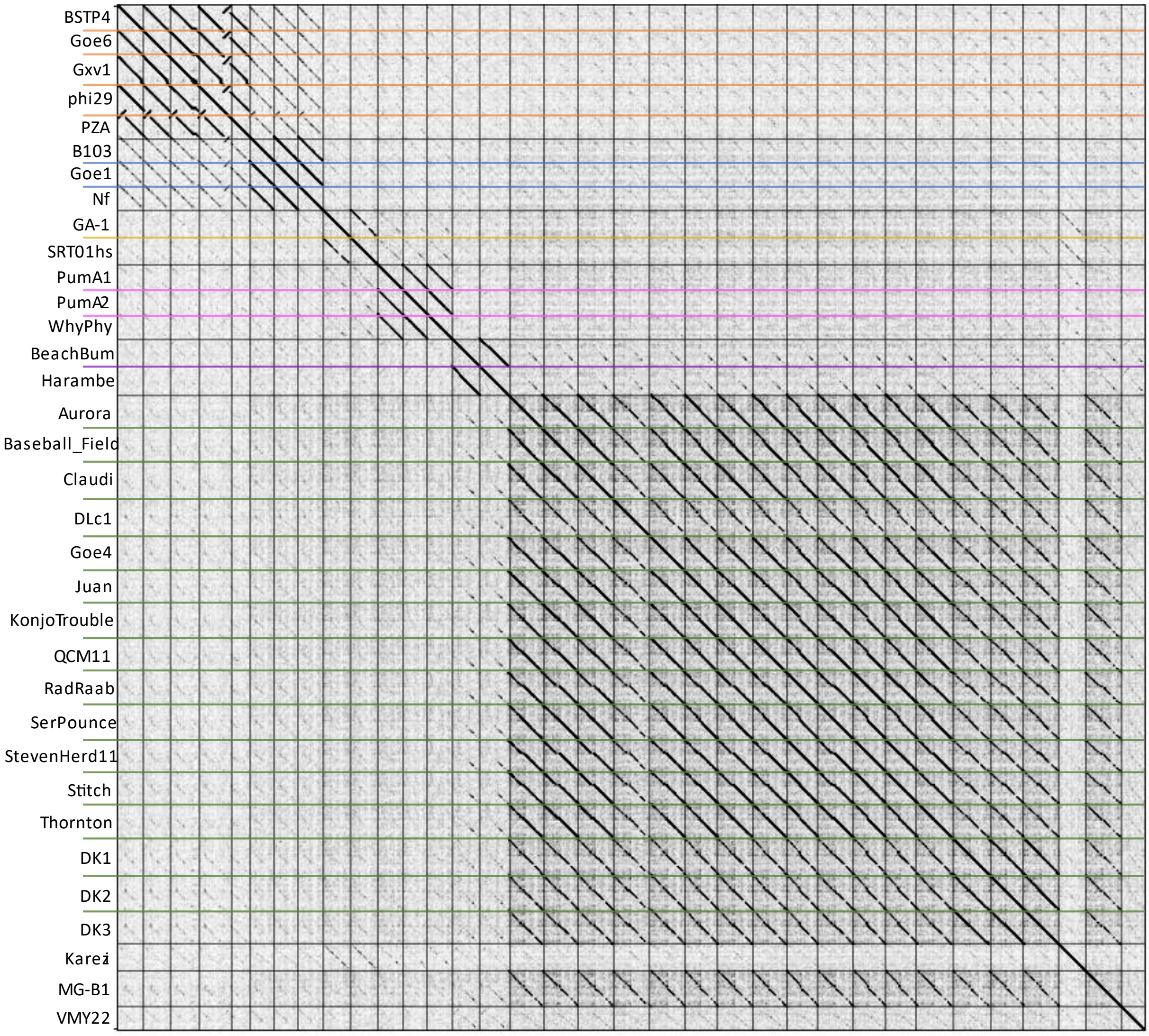

Figure S2. Whole genome dot plot of the *Salasmaviridae*/phi29-like phages. The phage genomes were aligned by BLASTn identities and then compared to each other over the x and y axis, producing a solid black line running down the centre displaying each phage against itself. Preliminary clusters are grouped by coloured lines if they appear to share >50% of their genomes.

Table S2. VIRDIC intergenomic similarities between clusters. Singleton phages are matched with their closest BLASTn result.

B1

	PZA	Gxvl	Goe6	phi29	BSTP4
PZA	100	88.75	89.18	89.69	89.20
Gxvl		100	92.39	92.55	91.64
Goe6			100	94.90	94.58
phi29				100	95.24
BSTP4					100

B4

	PumA1	PumA2	WhyPhy
PumA1	100	80.32	80.28
PumA2		100	85.19
WhyPhy			100

B2

	B103	Goe1	Nf
B103	100	88.57	90.28
Goe1		100	93.74
Nf			100

B5

	BeachBum	Harambe
BeachBum	100	93.71
Harambe		100

B3

	GA-1	SRT01hs
GA-1	100	65.65
SRT01hs		100

B7

	DK1	DK2	DK3
DK1	100	80.542	81.854
DK2		100	87.297
DK3			100

Singletons

	DLc1	Goe4	MG-B1	Stitch	Karezi	SRT01hs	VYM22	Harambe
DLc1	100	50.87						
Goe4		100						
MG-B1			100	49.41				
Stitch				100				
Karezi					100	38.30		
SRT01hs						100		
VYM22							100	23.86
Harambe								100

B6

	SerPounce	Claudi	KonjoTrouble	Aurora	Juan	QCM11	Goe4	Stitch	RadRaab	StevenHerd11	Baseball_field	Thornton
SerPounce	100	89.00	85.61	77.00	79.91	80.80	75.15	79.24	79.88	79.84	90.22	87.97
Claudi		100	91.06	78.42	82.41	83.00	75.89	81.28	81.23	81.18	91.67	89.83
KonjoTrouble			100	80.46	82.87	83.72	76.39	81.16	81.02	80.96	88.70	89.76
Aurora				100	83.21	83.13	78.64	77.19	78.80	78.77	79.10	77.22
Juan					100	84.55	82.75	81.85	81.47	81.41	81.99	81.134
QCM11						100	80.19	82.75	82.04	81.95	81.10	82.36
Goe4							100	80.26	81.84	81.87	74.30	75.45
Stitch								100	96.99	96.96	79.90	81.75
RadRaab									100	99.87	80	81.43
StevenHerd11										100	79.98	81.383
Basebal_fiield											100	88.25
Thornton												100

Table S3. *B. pumilus* mutant strains resistant to PumA1 and PumA2 infection.

Strain	Genome position	Mutation	Product
***B. pumilus* A2M1**	505, 868	c.505868_505869insTTTTA<br>p.M43IfsX29	TagF
***B. pumilus* A2M3**	507, 815	c.507815G>A<br>p.G688S	TagF
***B. pumilus* A2M7**	477, 569	c.477569delA<br>p.F237LfsX7	TagT
***B. pumilus* A2M11**	505, 868	c.505868_505871delTTTA<br>p.F42*fsX1	TagF
***B. pumilus* A2M14**	478, 245	c.478245delT<br>p.K12SfsX3	TagT
***B. pumilus* A1M3**	506, 177	c.506177delA<br>p.K143NfsX4	TagF
***B. pumilus* A1M5**	478, 262	c.478263_478295del<br>p.0	TagT
